# Supplementary material for: A Pilot Study of Short-Course Oral Vitamin A and Aerosolised Diffuser Olfactory Training for the Treatment of Smell Loss in Long COVID
Source: Brain Sci. 2023 Jun 30;13(7):1014. doi: 10.3390/brainsci13071014 (PMC10377650; doi:10.3390/brainsci13071014)
Supplement: Supplementary file 1 [file brainsci-13-01014-s001.zip › brainsci-2430896-SI.pdf]

Supplementary material

Table of Contents

SUPPLEMENTARY TABLES		
**Supplementary table 1.**	Vitamin A treatment in olfactory dysfunction	p. 3
**Supplementary table 2.**	Randomised clinical trials against COVID–19-related olfactory dysfunction	p. 4
**Supplementary table 3.**	Butanol threshold test	p. 5
**Supplementary table 4.**	Magnetic resonance (MR) spectroscopy at the gyrus rectus and superior frontal gyrus	p. 6
**Supplementary table 5.**	Volumetric measurements of the olfactory bulb and tract	p. 7
**Supplementary table 6.**	COVID–19 human autopsy studies evaluating the olfactory neuroepithelium and olfactory bulb and tract	p. 8
**Supplementary table 7.**	Animal studies investigating the pathophysiology of SARS-CoV-2 infection at the olfactory neuroepithelium and olfactory organs	p. 9–11

SUPPLEMENTARY FIGURES		
**Supplementary figure 1.**	Aerosolised diffuser olfactory training	p. 12
**Supplementary figure 2.**	Seed regions of the olfactory network	p. 13
**Supplementary figure 3.**	Three-dimensional (3D) representations of the primary and secondary olfactory cortical network (OCN) areas, created from 28 automatic anatomical labelling (ALL) regions	p. 14
**Supplementary figure 4.**	Interim (week 2) rs–fMRI brain scan images (left caudate nucleus as the seed region)	p. 15
**Supplementary figure 5.**	Correlations between functional connectivity in the left inferior frontal gyrus and end-of-treatment SIT scores	p. 16
**Supplementary figure 6.**	Correlations between magnetic resonance (MR) spectroscopy analysis and clinical olfactory function measurements at the end-of-treatment	p. 17

SUPPLEMENTARY MATERIALS		
**Supplementary material 1.**	Research protocol	p. 18–27

Supplementary table 1. Vitamin A treatment in olfactory dysfunction

Year	Author	Study design	Olfactory training	Vitamin A (VitA) treatment				Olfactory assessment(s)	Neuroimaging assessment	Clinical outcome
				Route (method)	Chemical compositions	VitA Regimen (IU)	Total VitA exposure (IU)			
**With olfactory training**										
2021	Chung TW, et al.[11]	Case report	Aerosolisation diffuser†	PO (capsule)	RP	25,000 IU per day, duration 2 weeks	350,000	BTT, SIT	Yes (rs-fMRI brain)	Uncertain
2017	Hummel T, et al.[12]	Retrospective study	Conventional‡	IN (drops)	RP	10,000 IU per day, duration 8 weeks	560,000	SST	Nil	Uncertain
**Without olfactory training**										
2017	Kartal D, et al.[13]	Prospective, non-controlled, non-randomised study	Nil	PO	Isotretinoin (13-cis-retinoic acid)	Not specified	Not specified	SST	Nil	Uncertain
2012	Reden J, et al.[14]	Double-blind, placebo-controlled, randomised trial	Nil	PO	Not specified	10,000 IU/day, duration 3 months	900,000	SST	Nil	No benefits
1962	Duncan RB, et al.[15]	Not specified	Nil	IMI, PO (tablet, emulsion)	Not specified	IMI 100,000 IU per week, duration 6 weeks; followed by PO 50,000 IU per day, duration 3–12 weeks	1,650,000	Self-reporting	Nil	Uncertain

†4 odours presented in four individual diffuser units, 20 seconds of odour exposure via individual aerosolisation diffusers, three training sessions per day, duration 4 weeks; ‡4 odours presented in four different glass jars, 10 seconds exposure from each jar, two training sessions per day, duration 12 weeks. BTT=butanol threshold test; IU=international units; IMI=intramuscular injection; IN=intranasal; PO=per oral; rs-fMRI=resting-state functional magnetic resonance imaging; RP=retinyl palmitate; SIT=smell identification test; SST=sniffin' sticks test.

Supplementary table 2. Randomised clinical trials against COVID–19-related olfactory dysfunction

Author (Country, date)	Subjects (n; female %)	Duration of OD at enrolment (days)	Nasoendoscopy examination	Objective olfactory measurement(s)	Interventions (n)	Olfactory training (OT)	Trial outcome(s)	Comment(s)
L. A. Vaira, et al.[18] (Italy; Oct 24, 2020)	18 (61%)	≥30 days	Nil	CCCRC<br>Threshold test: BTT<br>Identification test: culturally relevant odorants used	Corticosteroid group (n=9)<br>PO prednisone (1 mg/kg/day; tapering dose over 15 days) and nasal irrigation with betamethasone, ambroxol (mucolytic), and rinazine (decongestant).<br>Control group (n=9)<br>No active intervention	Nil	CCCRC test scores in the ccorticosteroid group was higher than the control group (p=0.024) at 40–day.	1. Nasoendoscopy examinations were not performed, alternative aetiologies of OD were not excluded, specifically allergic rhinitis and other atopies.<br>2. If nasoendoscopic evidence of atopy or allergic rhinitis were present, systemic and/or topical corticosteroid would likely improve olfactory outcome, regardless of COVID–19-related olfactory dysfunction.<br>3. Trial outcomes were not defined.<br>4. Details of nasal irrigation treatments were not specified.<br>5. The use of mucolytics and decongestants were major confounding factors in the interventional group.<br>6. Interventions did not include OT, which is the recognised standard-of-care for olfactory dysfunction.
A. A. Abdelalim, et al.[19] (Egypt; Jan 4, 2021)	100 (54%)	>7 days	Nil	Nil	Topical corticosteroid group (n=50)<br>Mometasone furoate nasal spray 100μg (2 puffs) once daily per nostril, in combination with OT for 3 weeks<br>OT group (n=50)<br>OT alone for 3 weeks	**Treatment duration**: 3 weeks<br>**Delivery method**: not specified<br>**Number of odorant(s) used**: 3 (rose, lemon, and clove)<br>**Treatment frequency**: 2 sessions per day<br>**Odorant exposure time**: 20s per session	At week 3, no statistically significant difference in the visual analog scale scores were found between the topical corticosteroid group and OT group (p=0.310).	1. Nasoendoscopy examinations were not performed, alternative aetiologies of OD were not excluded.<br>2. Objective olfactory function measurements were not performed.<br>3. Trial outcomes were not defined.
H. Kasiri, et al.[20] (Iran; Jun 8, 2021)	77 (49%)	≥14 days	Nil	Iran–SIT	Topical corticosteroid group (n=39)<br>Mometasone furoate 0.05% nasal spray 100μg, 2 puffs per nostril twice daily, in combination with OT for 4 weeks<br>Control group (n=38)<br>Topical sodium chloride nasal spray, 2 puffs per nostril twice daily, in combination with OT for 4 weeks	**Treatment duration**: 4 weeks<br>**Delivery method:** olfactory pen<br>**Number of odorant(s) used**: 1 (eucalyptus)<br>**Treatment frequency**: 2 sessions per day<br>**Odorant exposure time**: 20s per session	No statistically significant differences were found in the Iran–SIT scores at the end-of-treatment (p=0.239).	1. Nasoendoscopy examinations were not performed, alternative aetiologies of OD were not excluded.<br>2. Conflicting results were reported between the Iran–SIT scores and categorical differences in the severity of smell loss.
R. A. Rashid, et al.[21] (Iraq; Apr 7, 2021)	276 (72%)	4.5 (3–6)	Nil	Nil	Topical corticosteroid group (n=138)<br>Intranasal betamethasone sodium phosphate drops (0.1 mg/mL), 3 drops per nostril, 3 times per day, for 4 weeks<br>Placebo group (n=138)<br>Intranasal placebo drops (0.9% sodium chloride solution), 3 drops per nostril, 3 times per day, for 4 weeks	Nil	Compared to placebo group, intranasal betamethasone drops showed no significant effect on the recovery time of anosmia (hazard ratio 0.88; 95% CI 0.68–1.14; p=0.310).	1. Patients suffering from anosmia for >15 days were excluded.<br>2. Nasoendoscopy examinations were not performed, alternative aetiologies of OD were not excluded.<br>3. Objective olfactory function measurements were not performed.<br>4. Trial outcomes were not defined.<br>5. Interventions did not include OT, which is the recognised standard-of-care for olfactory dysfunction.
E. Yildiz, et al.[22] (Turkey; Jul 4, 2021)	150 (44%)	Not specified	Nil	Nil	Interventional group (n=50)<br>Saline irrigation (10 mL per nostril) twice daily per nostril, and triamcinolone acetonide 0.055% nasal spray 2 puffs per nostril twice daily, for 4 weeks<br>Comparator group (n=50)<br>Saline irrigation (10 mL per nostril) twice daily per nostril, for 4 weeks<br>Control group (n=50)<br>No active intervention	Nil	1. At 30–day, SROS of the interventional group was significantly higher than control (p=0.018) and comparator groups (p=0.033).<br>2. ODD was significantly reduced in the interventional group in comparison to the control (p=0.022) and comparator groups (p=0.028), at 30–day.	1. Nasoendoscopy examinations were not performed, alternative aetiologies of OD were not excluded.<br>2. Objective olfactory function measurements were not performed.<br>3. Trial outcomes were not defined.<br>4. Interventions did not include OT, which is the recognised standard-of-care for olfactory dysfunction.

BTT=butanol threshold test. CCCRC=Connecticut chemosensory clinical research center test. Iran-SIT=smell identification test (Iranian version). OD=Olfactory dysfunction. ODD=Olfactory Dysfunction Duration. OT=olfactory training. SROS=Self-Rating Olfactory Score.

Supplementary table 3. Butanol threshold test

Steps	Dilution steps	1-butanol (mL)	Deionized water (mL)	1-butanol concentration (%)
1	1	20mL of 99.9% 1-butanol	480	4.00000
2	2	50mL of dilution step 1	150	1.00000
3	3	50mL of dilution step 2	150	0.25000
4	4	50mL of dilution step 3	150	0.06250
5	5	50mL of dilution step 4	150	0.01563
6	6	50mL of dilution step 5	150	0.00391
7	7	50mL of dilution step 6	150	0.00098
8	8	50mL of dilution step 7	150	0.00024
9	9	50mL of dilution step 8	150	0.00006
10	10	50mL of dilution step 9	150	0.00002

10–steps serial dilution of 99.9% 1-butanol (AnalaR NORMAPUR®, VWR International limited, Briare, France). Each butanol threshold test (BTT) bottles contained 100mL of test solution, excess volume was discarded. Identical bottles containing deionized water were used as controls. All solutions were colourless. During the assessment, the test subjects were blindfolded. Each nostril was tested independently (untested nostril was occluded by a piece of sterile gauze), starting from the lowest concentration (0.00002% 1-butanol, step 10). Each concentration was tested along with a control in a forced-choice paradigm, in which the participant was required to differentiate between the test and control solutions. The step at which the participant was able to correctly differentiate between the test and controls solutions over four consecutive trials was the BTT score. The average BTT scores from both nostrils was the participant's final BTT score. If the participant failed to identify the highest concentration (4% 1-butanol, step 1), a BTT score of zero was awarded.

Supplementary table 4. Magnetic resonance (MR) spectroscopy at the gyrus rectus and superior frontal gyrus

	Intervention groups			
**Mean metabolite concentration ratios**	**Combination (n = 10)**	**Standard care (n = 9)**	**Control (n = 5)**	***P* value**
**Baseline (pre-treatment)**				
NAA/Cr	1.41±0.13	1.37±0.09	1.33±0.11	0.345
Cho/Cr	0.99±0.11	1.04±0.08	1.10±0.06	0.071
**Interim (week 2)**				
NAA/Cr	1.41±0.14[a]	1.40±0.13	1.24±0.06[a]	0.021*
Cho/Cr	1.00±0.09	1.09±0.10	1.08±0.07	0.050*
**End-of-treatment (week 4)**				
NAA/Cr	1.40±0.12[b]	1.38±0.09[c]	1.22±0.10[bc]	0.013*
Cho/Cr	0.98±0.07	1.07±0.09	1.06±0.08	0.068

Control=clinical observation. Standard care=OT alone. NAA/Cr=*N*-acetylaspartate/creatine ratio. Cho/Cr=Choline/creatine ratio. Combination=oral vitamin A in combination with aerosolised diffuser olfactory training (OT). Control=clinical observation. OT=olfactory training. Brown-Forsythe ANOVA with Tukey post hoc test: [a, b, c]$P < 0.050$. *$P < 0.050$.

Supplementary table 5. Volumetric measurements of the olfactory bulb and tract

	Intervention groups (n)			
	**Combination (*n* = 10)**	**Standard care (*n* = 9)**	**Control (*n* = 5)**	***P* value**
**Baseline (pre-treatment)**				
Left side (mm^3)	68.66±16.10	65.85±25.08	65.24±10.33	0.920
Right side (mm^3)	70.62±20.89	66.73±25.33	67.25±21.58	0.920
**End-of-treatment (week 4)**				
Left side (mm^3)	70.00±19.30	75.86±29.57	72.33±15.15	0.840
Right side (mm^3)	74.68±22.00	76.64±31.22	75.11±19.23	0.980

Combination=oral vitamin A in combination with aerosolised diffuser olfactory training (OT). Standard care=OT alone. Control=clinical observation. Brown-Forsythe ANOVA with Tukey post hoc test.

Supplementary table 6. COVID–19 human autopsy studies evaluating the olfactory neuroepithelium and olfactory bulb and tract

Author (Sample size, *n*)	Methods of analysis	Olfactory neuroepithelium (ONE)	Olfactory bulb (OB) and tract
D Kirschenbaum, et al.[34] (*n* = 2)	1. Routine histological staining<br>2. IHC staining	1. Prominent leukocytic infiltrates in the lamina propria and focal atrophy of the ONE<br>2. IHC revealed a slight predominance of $CD3^+$ T cells over $CD20^+$ B lymphocytes	Olfactory tracks were unremarkable
J Matschke, et al.[35] (*n* = 43)	1. Routine histological staining<br>2. IHC staining[a]<br>3. qRT–PCR (E gene)<br>4. Human brain single-cell transcriptome analysis[b]	Not assessed	1. High degree of astrogliosis ($GFAP^+$) and microgliosis ($HLA\text{-}DR^+$ and $IBA1^+$)<br>2. Mild infiltration by cytotoxic T lymphocytes ($CD8^+$)<br>3. In-silico analysis of neurons, glial cells, and endothelial cells showed genetic expressions of *ACE2*, *TMPRSS2*, *TPCN2*, *TMPRSS4*, *NRP1*, and *CTSL*, supporting their capacity for SARS-CoV-2 viral entry and infection
J Meinhardt, et al.[36] (*n* = 33)	1. Routine histological staining<br>2. IHC staining[c]<br>3. qRT–PCR (E gene, sgRNA)<br>4. Electron microscopy (EM)<br>5. Cytokine profiling<br>6. RNAScope ISH for the detection of messenger RNA (V-ncoV2019-S probe)	1. ACE2 detected by IHC<br>2. High SARS-CoV-2 viral load detected by qRT–PCR, furthermore SARS-CoV-2 sgRNA detected in four out of 20 (20%) ONE samples<br>3. Selective EM assessment of ONE region which showed strong SAR-CoV-2 RNA ISH signals<br>4. Colocalisation studies revealed perinuclear SARS-CoV-2 S protein immunoreactivity in $TuJ1^+$, $NF200^+$, and OMP^+ neuronal cells in three COVID–19 patients	1. No reliable detection of ACE2 by IHC staining<br>2. SARS-CoV-2 RNA detected in three out of 32 (9%) olfactory bulb specimens<br>3. sgRNA not detectable at the OB
B Schurink, et al.[37] (*n* = 21; brain autopsy, *n* = 9)	1. Routine histological staining<br>2. IHC staining, including CD3, CD4, CD8, GFAP, HLA–DR, and OMP	Not assessed	1. Extensive inflammation at the OB and medulla oblongata<br>2. All patients (*n* = 9) showed activation and clustering of microglia ($HLA\text{-}DR^+$), astrogliosis ($GFAP^+$), and perivascular cuffing of T cells ($CD3^+$)

COVID–19=coronavirus disease 2019. E gene=envelope gene. IHC=immunohistochemical. ISH=in situ hybridisation. qRT–PCR=quantitative reverse transcription–polymerase chain reaction. SARS-CoV-2=severe acute respiratory syndrome coronavirus 2. sgRNA=subgenomic RNA. [a]CD68 (marker for phagocytic activity). CD8 (cytotoxic T lymphocyte). GFAP=antibodies against human glial fibrillary acidic protein (astrocyte marker). HLA–DR=Human Leukocyte Antigen–DR (activated microglia marker). IBA1=ionized calcium-binding adaptor molecule 1 (microglia activity marker). SARS-CoV-2 N protein=antibody against viral nucleocapsid (N) proteins. SARS-CoV-2 S protein=antibody against viral spike (S) proteins. TMEM119=transmembrane protein 119. [b]*ACE2*=angiotensin-converting enzyme 2. *CTSL*=cathepsin L. *NRP1*=neuropilin 1. *TMPRSS2*=transmembrane serine protease 2. *TMPRSS4*=transmembrane serine protease 4. *TPCN2*=two pore segment channel 2. [c]ACE2. AE1/AE3 (pan Cytokeratin antibody). CD56 (neural cell adhesion molecule). MRP14 (Myeloid-Related Protein-14). NF200 (neurofilament-200). OLIG2 (oligodendrocyte transcription factor 2). OMP (olfactory marker protein). S100. SARS-CoV-2 spike glycoprotein. βIII tubulin (TuJ1, class III β-tubulin).

Supplementary table 7. Animal studies investigating the pathophysiology of SARS-CoV-2 infection at the olfactory neuroepithelium and olfactory organs

**Author**	**Animal characteristics**	**Experiment procedure**	**Methods of analysis**	**Olfactory neuroepithelium (ONE)**	**Olfactory bulb (OB) and tract**
**Golden Syrian hamsters**					
B Bryche, et al.[38]	Animals (*n* = 12)<br>Female<br>8 weeks	Intranasal infection<br>SARS-CoV2 strain UCN1 (5.4×10^3 PFU) infected animals (*n* = 5)<br>SARS-CoV2 strain UCN19 (1.8×10^3 PFU) infected animals (*n* = 5)<br>Mock-infected animals (*n* = 2)<br>Necropsy examination<br>DPI: 2, 4, 7, 10 or 14	1. Routine histological staining<br>2. IHC staining[a]<br>3. qRT–PCR	1. Significant histological damage and disorganisation of the ONE (2 DPI)<br>2. Evidence of SARS-CoV-2 infection of the SUS cells (CK18$^+$), but not mature OSN (OMP$^+$)<br>3. Diminished G_{olf} signals at the ONE in SARS-CoV-2 infected animals from 2–10 DPI, indicated cilia lost<br>4. Morphologically activated IBA1$^+$ cells at the ONE and lamina propria, demonstrating immune cells infiltration	SARS-CoV-2 was not detected at the OB
AJ Zhang, et al.[33]	Animals (*n* = 18)<br>Female & male<br>6–8 weeks	Intranasal infection<br>1×10^5 PFU SARS-CoV-2 virus HKU-001a strain infected animals (*n* = 15)<br>Mock-infected animals (*n* = 3)<br>Necropsy examination (3 animals per time points)<br>HPI: 12<br>DPI: 2, 4, 7, 14	1. Routine histological staining<br>2. IHC staining[b]<br>3. TUNEL staining<br>4. qRT–PCR (RdRp gene)<br>5. Viral culture (VeroE6)<br>6. Cytokine profiling	1. Peak viral load (4 DPI) and infectious viral titre ($TCID_{50}$, 2 DPI) were found in the ONE<br>2. Extensive and diffuse distribution of SARS-CoV-2 N protein across the ONE at 2 & 4 DPI, with associated ONE desquamation<br>3. Extensive SUS cell infections were documented morphologically.<br>4. Neuronal infection by SARS-CoV-2 was demonstrated by double immunofluorescence staining, revealed colocalisation of SARS-CoV-2 N protein, mature OSN (OMP$^+$), and immature OSN (Tuj1$^+$)<br>5. ONE apoptosis was confirmed by TUNEL staining<br>6. Significant expression of proinflammatory cytokine and chemokine genes were detected after SARS-CoV-2 infection	SARS-CoV-2 was not detected at the OB
**K18-hACE2 transgenic mice**					
J Zheng, et al.[39]	Animals (*n* = 24)<br>Female &<br>male	Intranasal infection<br>2019n-CoV/ USA-WA1/2019 strain infected animals:	1. Routine histological staining<br>2. IHC staining	1. SARS-CoV-2 antigen was readily detected at 2 & 5 DPI<br>2. Loss of cellularity at the ONE by 5 DPI	SARS-CoV-2 N protein staining was detected at 6

	7–8 weeks	1×10^{3} PFU (n = 3),<br>1×10^{4} PFU (n = 10), and<br>1×10^{5} PFU (n = 8)<br>Mock-infected animals (n = 3)<br>Necropsy examination (performed for the 1×10^{5} PFU infected group)<br>DPI: 2, 4, 5, 6	3. qRT–PCR<br>4. Viral culture (VeroE6)<br>5. Functional olfactory assessments[c]	3. SARS-CoV-2 antigen was detected in SUS cells	DPI (2 out of 3 mice) in the OB
**Ferrets**					
HE Everett, et al.[40]	Animals (n = 12)<br>Female<br>~5 months	Intranasal infection<br>1.2×10^{6} $TCID_{50}$/mL SARS-CoV-2/Australia/VIC01/2020 infected animals (n = 12)<br>At 21 days, 2 ferrets were rechallenged with 2.0×10^{6} $TCID_{50}$/mL of the same strain<br>Necropsy examination (2 animals per time points)<br>DPI: 3, 5, 7, 14, and 21<br>DPI: 24 (for 2 ferrets which received rechallenge)	1. Routine histological staining<br>2. IHC staining<br>3. qRT–PCR<br>4. Viral culture (VeroE6)<br>5. RNAScope ISH	1. IHC demonstrated SARS-CoV-2 S and N antigen within the ONE; in particular, S antigens were localised at the apical surface of the ONE<br>2. SARS-CoV-2 N proteins were present in various cell populations identified by morphology: SUS cells, OSN, and neuronal tract within the lamina propria of the ONE<br>3. RNAScope ISH demonstrated presence of SARS-CoV-2 RNA in the SUS cells and OSN	SARS-CoV-2 was not detected at the OB
**Rhesus monkeys**					
L Jiao, et al.[41]	Animals (n = 9)<br>Sex (NS)<br>3–5 years<br>3–5 Kg	SARS-CoV-2 viral stock was provided by the GPCDC[d]<br>Experimental infection<br>Intranasal injection<br>1×10^{7} PFU (n = 5)<br>Intracranial injection<br>1×10^{5} PFU (n = 1)<br>1×10^{6} PFU (n = 1)<br>Mock-infected animals<br>Intranasally and intracranially PBS injection (n = 1)<br>Untreated control (n = 1)<br>Necropsy examination (for intranasally infected animals)<br>DPI: 1 (n = 1), 4 (n = 1), 7 (n = 1), 14 (n = 2)	1. Routine histological staining<br>2. IHC staining<br>3. qRT–PCR<br>4. Viral culture (VeroE6)	1. After high-dose intranasal infection, SARS-CoV-2 RNA was detectable in the ONE from 1–7 DPI<br>2. SARS-CoV-2 RNA was detectable at the olfactory trigone at 4 DPI	SARS-CoV-2 P antigens were detected in the OB (1 DPI)

DPI=days post-infection. HPI=hours post-infection. IHC=immunohistochemical. ISH=in situ hybridisation. NS=not specified. OB=olfactory bulb. OSN=olfactory sensory neuron. PBS=phosphate buffer solution. PFU=plaque forming units. qRT–PCR=quantitative reverse transcription–polymerase chain reaction. SARS-CoV-2 E gene=envelope gene. SARS-CoV-2 RdRP protein=RNA-dependent RNA polymerase. SARS-CoV-2 S protein=spike protein. SARS-CoV-2=severe acute respiratory syndrome coronavirus 2. SUS=sustentacular cells. $TCID_{50}$=Tissue Culture Infectious Dose 50 assay. TUNEL=Terminal deoxynucleotidyl transferase dUTP nick end labelling. [a]CK18=cytokeratin-18. G_{olf}=G_s subfamily of G_α subunits: $G_{\alpha\ s}$ and $G_{\alpha\ olf}$ for olfactory signal transduction. IBA1=ionized calcium-binding adaptor molecule 1 (monocyte and macrophage cell-specific marker). OMP (olfactory marker protein). SARS-CoV-2 N protein=antibody against viral nucleocapsid (N) proteins. [b]ACE2=angiotensin-converting enzyme 2. OMP (marker for mature OSN). SARS-CoV-2 N protein. Tuj1=neuron-specific class III β-tubulin (marker for immature OSN). [c]Abnormal functional olfactory assessments: social scent-discrimination assays (male mice); familiar and novel bedding assessments (female); buried food tests (female & male). [d]GPCDC=Guangdong Provincial Center for Disease Control and Prevention.

Supplementary figure 1. Aerosolised diffuser olfactory training

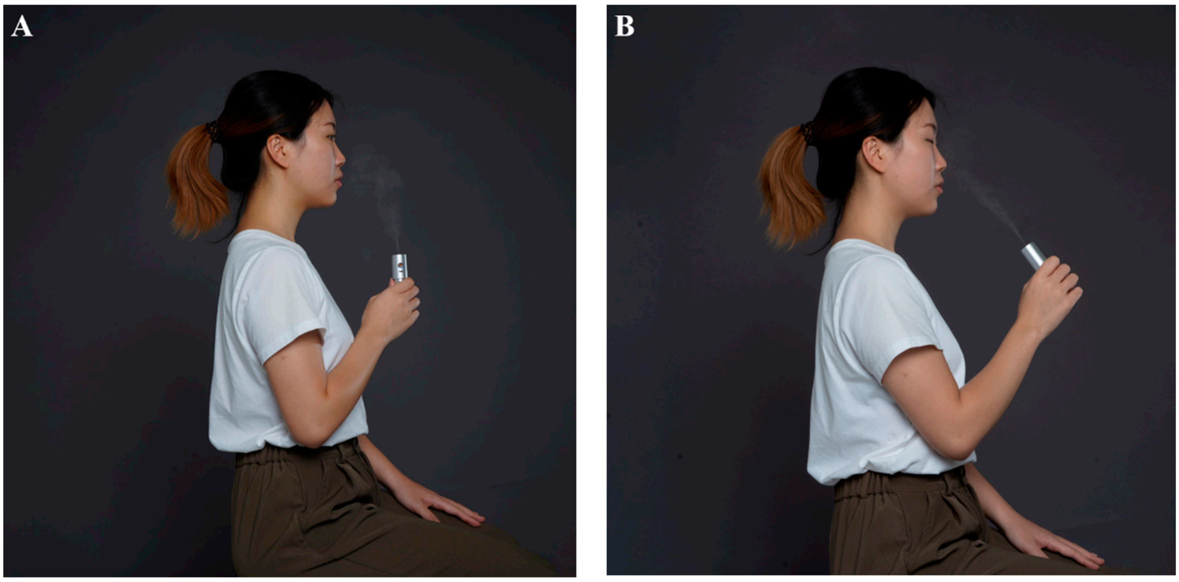

Aerosolised diffuser olfactory training. (A) Correct usage, stream of aerosolised essential oil directed vertically upwards, away from the user's face. (B) Incorrect usage, stream of aerosolised essential oil directed at the user's face, which may cause transient irritation to the skin and mucosal surfaces.

Supplementary figure 2. Seed regions of the olfactory network

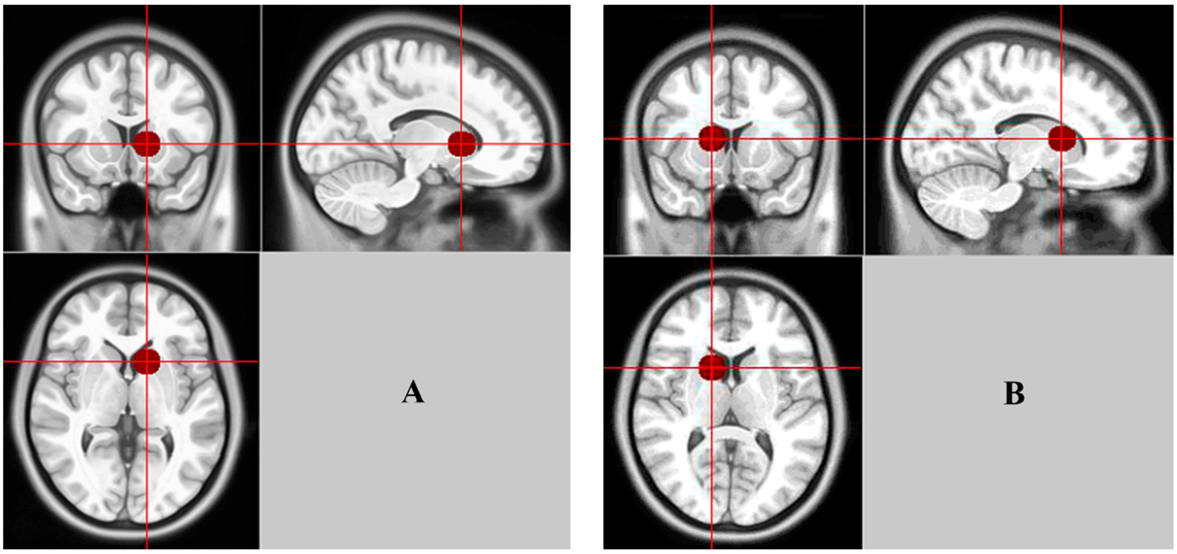

Seed regions of the olfactory network. (A) Left caudate nucleus. (B) Right caudate nucleus.

Supplementary figure 3. Three-dimensional (3D) representations of the primary and secondary olfactory cortical network (OCN) areas, created from 28 automatic anatomical labelling (ALL) regions

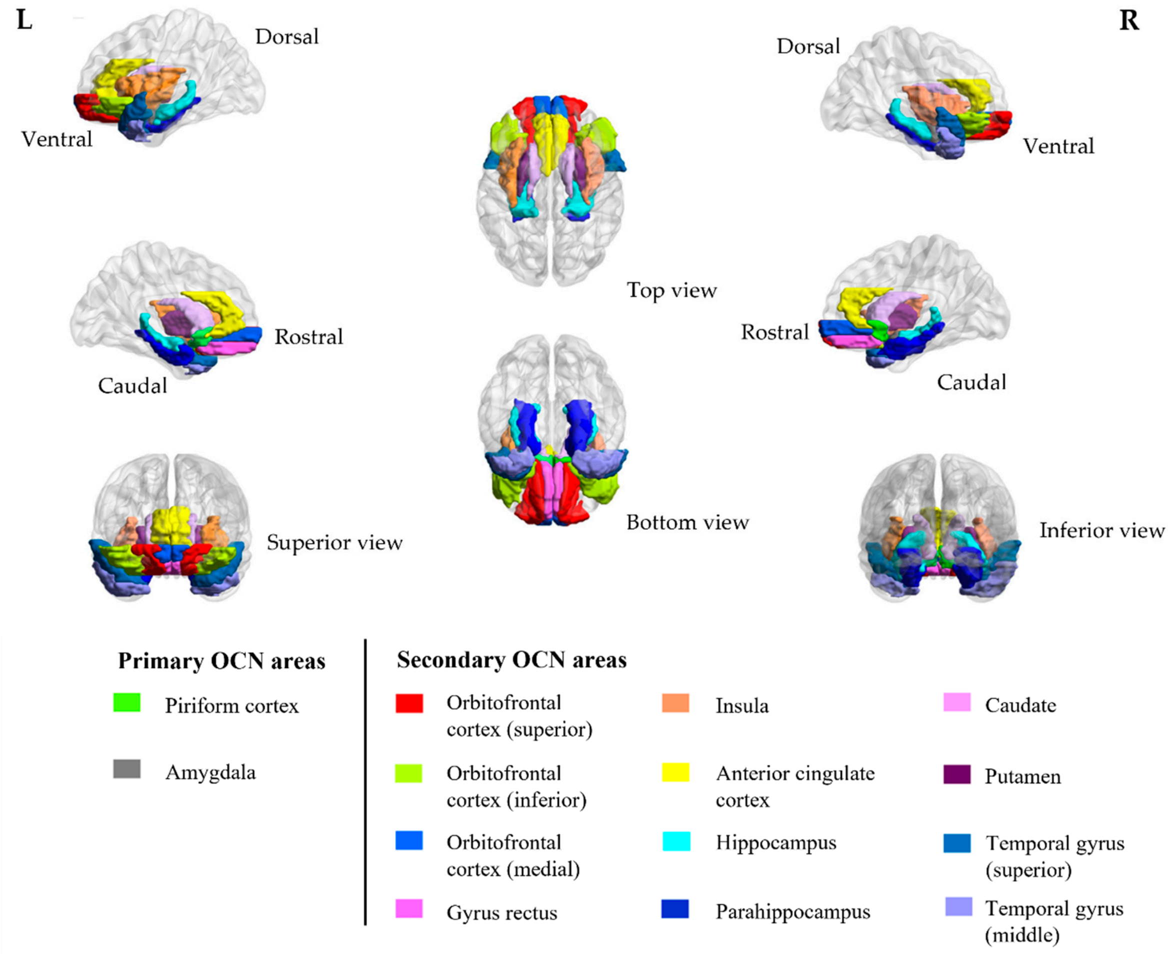

Supplementary figure 4. Interim (week 2) rs–fMRI brain scan images (left caudate nucleus as the seed region)

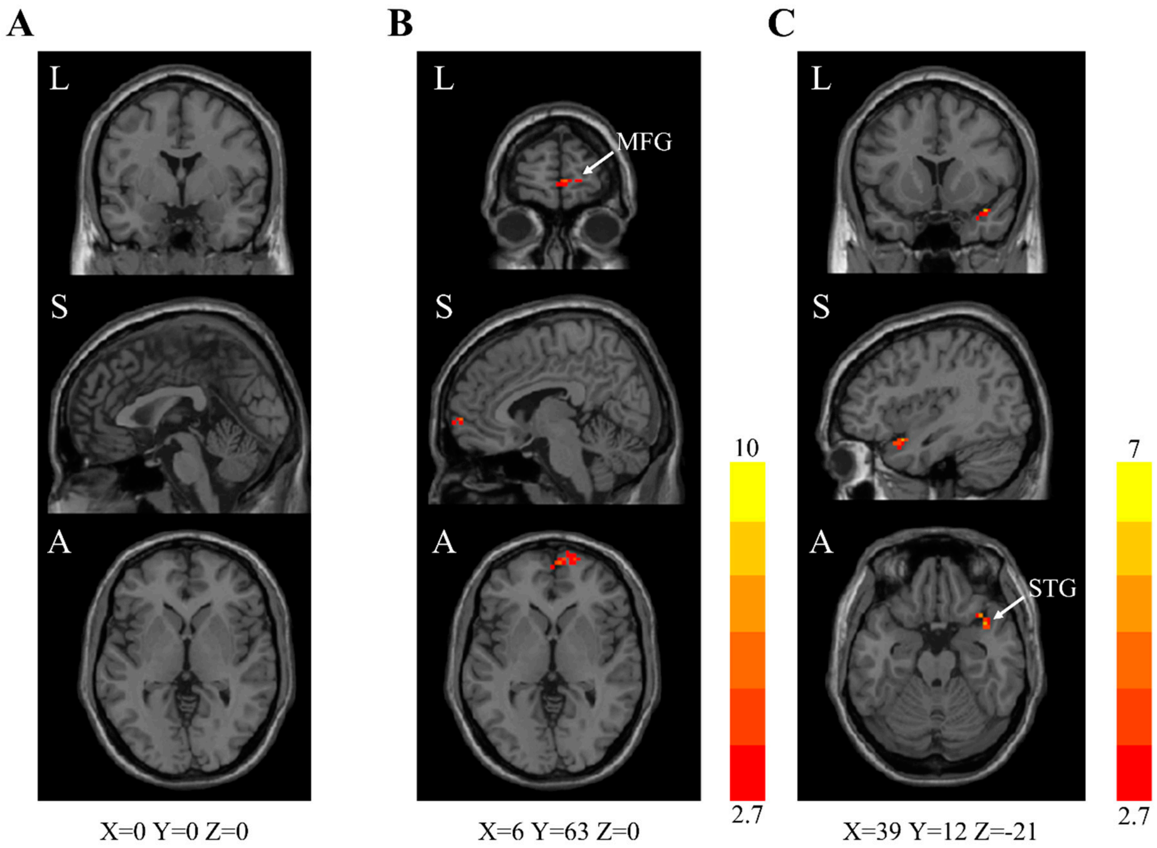

Two-sample *t* test. GRF correction (voxel-level $P < 0.010$, cluster-level $P < 0.050$). rs–fMRI=resting-state functional magnetic resonance imaging. z values are represented by the colour bars. A=anterior. L=left. S=superior. MFG=medial frontal gyrus (right side). STG=superior temporal gyrus (right side) (A) Combination [oral vitamin A in combination with olfactory training (OT)] group versus standard care (OT alone) group. (B) Combination group versus control (clinical observation) group. (C) Standard care group versus control group.

Supplementary figure 5. Correlations between functional connectivity in the left inferior frontal gyrus and end-of-treatment SIT scores

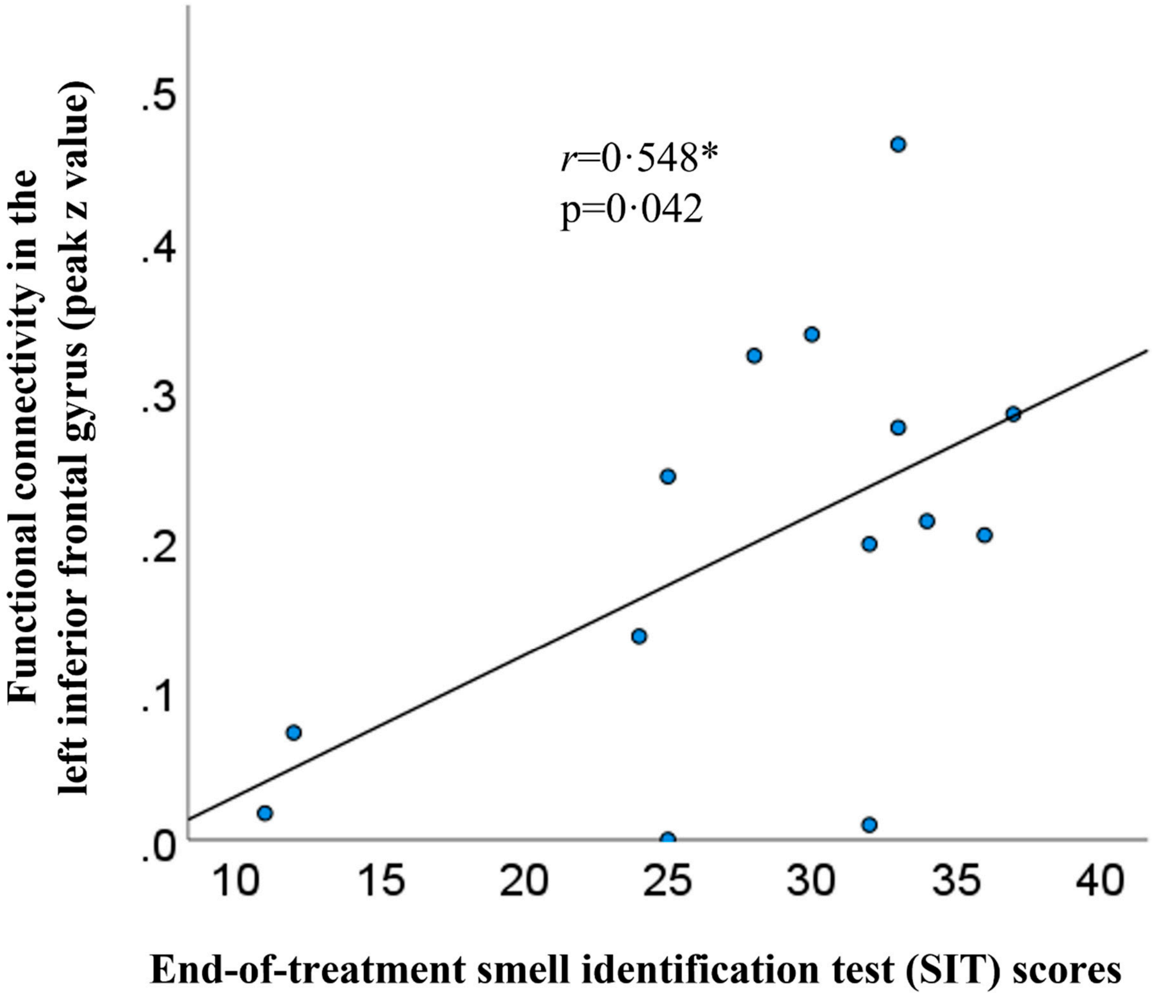

The peak z value represents the maximal functional connectivity in the left inferior frontal gyrus (between left caudate nucleus seed and voxels) for the combination and control group at the end-of-treatment [two-sample *t* test; GRF correction (voxel-level $P < 0.010$, cluster-level $P < 0.050$)], which demonstrated positive correlation with the smell identification test (SIT) scores ($n = 14$, $r = 0.548$, $P < 0.042$). $^{*}P < 0.050$.

Supplementary figure 6. Correlations between magnetic resonance (MR) spectroscopy analysis and clinical olfactory function measurements at the end-of-treatment

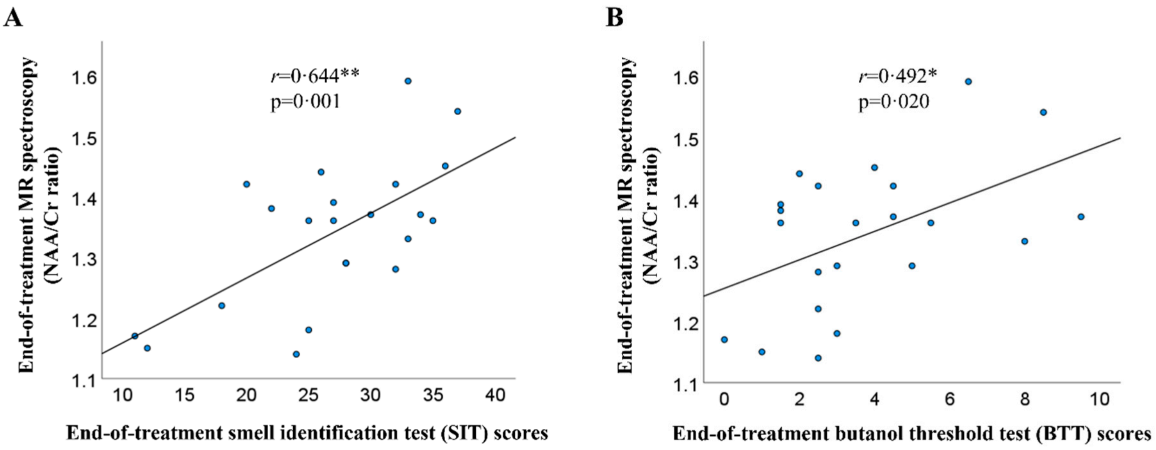

Positive correlations were found between the *N*-acetylaspartate/creatine (NAA/Cr) ratios measured at the gyrus rectus and superior frontal cortex by MR spectroscopy and the (A) smell identification test (SIT) scores (n = 22, r = 0.644, P = 0.001) and (B) butanol threshold test (BTT) scores (n = 22, r = 0.492, P = 0.020) at the end-of-treatment. *P < 0.050. **P < 0.010.

Supplementary material 1. Research protocol

PROTOCOL TITLE

Olfactory and neurosensory rehabilitation in coronavirus 2019 (COVID–19)-related olfactory dysfunction (OD)

Principal Investigators

Dr. Tom Wai-Hin Chung
Dr. Fergus Kai-Cheun Wong
Dr. Hui Zhang
Dr. Henry Ka-Fung Mak
Professor Ivan Fan-Ngai Hung

Date: 14 September 2020
Version: 8

Table of contents

1.	Protocol synopsis	p 20–21
2.	Clinical section	p 21
2.1	Background and rationale	p 21
2.1.1	Introduction	p 21–22
2.1.2	Background of the study article	p 22
2.1.2.1	Olfactory assessments	p 22
2.1.2.2	Ear, nose, and throat assessments	p 22
2.1.2.3	Olfactory training	p 22
2.1.2.4	Vitamin A	p 22–24
2.1.2.5	Resting-state functional magnetic resonance imaging (rs–fMRI) brain scans	p 24–25
3.1	Study hypothesis and objectives	p 25
3.1.1	Study hypothesis	p 25
3.1.2	Primary objectives	p 25
3.1.3	Secondary objectives	p 25
3.2	Selection of study population	p 25
3.2.1	Inclusion criteria	p 25
3.2.2	Exclusion criteria	p 25
3.3	Study design and organization	p 26
3.3.1	Overall study design	p 26
3.3.2	Intervention and control	p 26
3.3.3	Randomisation and blinding	p 26
3.3.4	Withdrawal	p 26
3.4	Outcome measurements	p 26
3.4.1	Primary outcome measurements	p 26
3.4.2	Secondary outcome measurements	p 26–27
4.1	Informed consent	p 27
4.2	Confidentiality of data	p 27
4.2.1	Confidentiality of study data	p 27
4.2.2	Confidentiality of subject data	p 27
4.2.3	Archive of data	p 27
4.3	Ethical and administrative endorsement	p 27
4.3.1	Administrative and ethical approval	p 27
4.3.2	Study team	p 27
5.	References	p 27–28

1. Protocol synopsis

Protocol title

Olfactory and neurosensory rehabilitation in coronavirus 2019 (COVID–19)-related olfactory dysfunction (OD).

Hypothesis

A combination of oral vitamin A (VitA) and intense aromatic chemosensory olfactory training (OT) by aerosolisation diffusers will expedite the neurosensory recovery of olfaction in patients suffering from prolonged COVID–19-related OD.

Primary objective

To evaluate the safety and efficacy of oral VitA in combination with OT, compared to OT alone, for the treatment against prolonged COVID–19-related OD.

Subject/patient definition

Recruited subjects include clinically recovered COVID–19 adult patients (≥18 years of age) complaining of prolonged (≥3 months) COVID–19-related OD, who are receiving follow-up care in Queen Mary Hospital (QMH) and Pamela Youde Nethersole Eastern Hospital (PYNEH) from June 2020 onwards. Written informed consents were obtained from all recruited subjects. Subjects must be available to complete the study and comply with study procedures.

Study design

This is an open–labeled, randomised-controlled trial (RCT), investigating the safety and therapeutic efficacy of oral VitA in combination with OT for patients suffering from prolonged COVID–19-related OD.

Prior to the initiation of treatment, all patients will receive objective olfactory assessessments. Comprehensive ear, nose, and throat (ENT) examination will be performed to rule out alternative causes of OD. All participants will receive resting-state functional magnetic resonance imaging (rs-fMRI) of the brain before treatment as baseline evaluation.

Patients with prolonged COVID–19-related OD will be assigned to the intervention (group A or B) or control (group C) arms:

Intervention arm

A. Short-course (14 days) daily oral VitA 25,000 international units (IU) in combination with OT three times per day for 4 weeks; or
B. OT three times per day for 4 weeks alone; or

Control arm

C. Clinical observation for 4 weeks

At the completion of the trial, objective olfactory assessessments will be repeated to document clinical changes in olfaction. Follow-up rs–fMRI will be performed to document neuroradiological changes in the brain structures and cerebral network functional connectivies (FC).

Intervention arm

A. Short-course (14 days) daily oral VitA 25,000 international units (IU) in combination with OT three times per day for 4 weeks; or
B. OT three times per day for 4 weeks alone

Control arm

Clinical observation for 4 weeks

Primary outcome

Objective changes in olfactory measurements between baseline and at 4 weeks, as documented by:

1. Butanol threshold test (BTT)
2. Smell identification test (SIT)

Secondary outcome

Documentation of objective modulations in the neuroradiological changes in the brain structures and cerebral network FC in the rs-fMRI brain scans between baseline, week 2, and week 4, as documented by:

1. Group independent component analyses and functional connectivity analyses of the olfactory, gustatory, somatosensory, and integrative networks before and after treatment in COVID–19 patients with OD
2. Group independent component analyses and functional connectivity analyses of the olfactory, gustatory, somatosensory, and integrative networks between combined VitA and OT versus OT alone in COVID–19 patients with OD
3. Group independent component analyses and functional connectivity analyses of the olfactory, gustatory, somatosensory, and integrative networks in the interventional versus control arms of COVID–19 patients with OD

Data analysis

Functional olfactory assessments: BTT and SIT scores between interventional and control arms will be compared using Student's *t* test. The BTT and SIT follow-up results for the interventional arm will be compared using paired *t* test. Categorical variables will be compared using the Fisher's exact test. SPSS 21.0 for Windows (SPSS Inc., IBM) will be used for statistical computation. $p<0.050$ represents significant difference.

rs-fMRI assessment: After the pre-process of the rs-fMRI data (DPABI, http://rfmri.org/dpabi), the analysis of data will be performed using Group ICA Of fMRI Toolbox (GIFT). Olfactory, gustatory, somatosensory, and integrative networks will be identified from 25 group-level spatial intrinsic connectivity maps. Two sample *t* test will be used to calculate the significance between two groups (FDR<0.05, cluster size>270mm^3). The relationship between clinical features and peak value of network FC will be estimated based on the Pearson correlation method in SPSS package (SPSS Inc., Chicago, USA).

2. Clinical section

2.1 Background and rationale

2.1.1 Introduction

Coronavirus disease 2019 (COVID–19) was declared a global pandemic by the World Health Organization on 11th March 2020. COVID–19 is caused by a novel betacoronavirus: severe acute respiratory syndrome coronavirus 2 (SARS-CoV-2). SARS-CoV-2 is highly adapted to humans and is capable of efficient person-to-person transmission.[1] As of 9th September 2020, COVID–19 has already infected 27 million people worldwide, with over 890,000 associated deaths.[2] Due to inadequacies in testing programs and large proportions of mild infections, the exact community burden of COVID–19 is unknown and will require population-wide seroepidemiological studies for definitive elucidation.

The common symptoms of COVID–19 include fever, cough, dyspnoea, malaise, myalgia, and gastrointestinal disturbances.[3] Most recently, our group has identified and confirmed that olfactory dysfunction (OD) is a common extra-pulmonary manifestation in COVID–19. In this study, OD was confirmed quantitatively using the butanol threshold test (BTT) and smell identification test (SIT). Mean BTT score of COVID–19 patients were worse when compared to healthy controls (p=0.004, difference in means=1.8; 95% CI: 0.6 – 2.9).[4]

In the evaluation of the aetiology of OD in COVID–19 patients, co-infection by other common respiratory viruses were excluded by polymerase chain reactions (PCR). Conductive impairments due to nasal polyps, sinusitis, and olfactory cleft obstruction were absent in most patients on nasoendoscopy and computed tomography scans of the sinuses. Finally, nasal biopsies were performed which demonstrated the presence of infiltrative CD68$^+$ macrophages harbouring SARS-CoV-2 antigen in the stroma. Therefore, our group postulates that COVID–19 -related OD may be due to direct invasion of the human olfactory neuroepithelium by SARS-CoV-2, in particular affecting the sustentacular and horizontal basal cells, which express the angiotensin converting enzyme 2 (ACE2) and transmembrane serine protease 2 (TMPRSS2) genes.[5]

Currently, effective treatment against COVID–19-related OD is lacking. Although most patients report subjective recovery of olfaction within two weeks, subtle impairments may be neglected which may negatively affect their quality of life. Importantly, there is a subgroup of COVID–19 patients who complain of persistent and profound OD despite clinical recovery.

Previous studies have demonstrated that olfactory training (OT) improves olfactory function in patients suffering from post-viral and post-traumatic olfactory loss.[6,7] The neuromodulatory effects of OT have also been demonstrated by functional magnetic resonance imaging (fMRI) of the brain.[8,9]

Vitamin A (VitA) treatment may contribute to the homeostasis of the olfactory system.[10] This theory was supported by a recent study which reported that the combination of topical intranasal vitamin A of 10,000 international units (IU) per day and OT for 8 weeks, produced a significantly greater olfactory improvement in patients with post-infectious and post-traumatic smell disorder, when comparing to OT alone.[11] Although, the safety profile of topical intranasal vitamin A treatment is

unknown, the safety and therapeutic effects of oral vitamin A in the reduction of morbidity and mortality in children with measles infection is well-established.[12–14]

Therefore, we propose to conduct an open-labelled randomized-controlled trial (RCT) to investigate the safety and therapeutic effects of a 14 days short-course of oral VitA 7500µg RAE (retinol activity equivalents) daily in combination with pulse aromatic chemosensory OT via aerosolisation diffusers three times per day for 4 weeks in the treatment against prolonged COVID–19 -related OD.

2.1.2 Background of the study articles

2.1.2.1 Olfactory assessments

All participants will receive quantitative olfactory function assessments:

1. Butanol threshold test (BTT)
2. Smell identification test (SIT)

2.1.2.2 Ear, nose, and throat assessments

All participants will receive complete ear, nose, and throat (ENT) examination plus fibreoptic laryngoscope examination. The endoscopic findings will be documented according to the Modified Lund–Kennedy (MLK) scoring system.[15]

2.1.2.3 Olfactory training

Olfactory training (OT) is a form of neurochemical stimulation using aromatic substances as a form of non-pharmacological olfactory rehabilitation in patients with post-infectious or post-traumatic olfactory dysfunctions.

Aromatic substances

The aromatic substance used during OT will be in the form of essential oils (Table 1). They will consist of four different aromatic categories, which are commonly encountered in the local Hong Kong population: fruits (i.e., lemon), shrubs (i.e., eucalyptus), flower (i.e., geranium,), and pine wood (i.e., cedarwood).

Table 1. Aromatic substances used in olfactory training

Common name	Botanical nomenclature
Lemon	*Citrus limon*
Eucalyptus	*Eucalyptus radiata*
Geranium	*Pelargonium graveolens*
Cedarwood	*Juniperus virginiana*

Intervention

As described above, the patient will be challenged with four different types of aromatic substances during OT. The odorants will be delivered via a novel electronic portable aromatic rehabilitation (EPAR) diffuser. Patients were instructed to conduct OT at a seated position, where the EPAR diffuser was placed at an arm's length with the stream of aerosolized essential oil directed upwards. During OT sessions, all four categories of essential oils were delivered via the EPAR diffusers for 20 seconds sequentially, providing a total of 80 seconds of olfactory stimulation three times per day.

Interactions and side effects

The included aromatic substances for the purpose of smell training are safe and without significant systemic health effects.

Contraindications

Hypersensitivity to aromatic substances are absolute contraindications.

2.1.2.4 Vitamin A

In measles infection, vitamin A treatment reduces morbidity and mortality in children.[12,13] Two doses of oral VitA [200,000 international units (IUs) on two consecutive days, 24 hours apart], which is equivalent to 400,000 IU of VitA has been shown to reduce the mortality of measles infection in children (< 2 years) and pneumonia-specific mortality.[14]

In terms of olfactory neurogenesis, it had been proposed that retinoic acid (RA), the active metabolite of VitA, plays an important regulatory role in the differentiation of olfactory neural progenitor cells

in vitro.[16] In human, intranasal VitA has been shown to provide superior clinical benefit when combined with OT in the treatment of olfactory loss.[11]

Preparation
Vitamin A (soft gels) 25,000 IU (7,500 µg RAE; Carlson Laboratories, Illinois, USA).

Standardised unit of measurements for vitamin A
The standardized unit of conversions for the measurement for vitamin A, according to the Food and Drug Administration [FDA (U.S.)] are outlined in Table 2.[17]

Table 2. Standardized unit conversion of vitamin A

1 microgram (µg) RAE	= 1 µg pre-formed vitamin A (retinol)
	= 2 µg supplemental β-carotene
	= 12 µg dietary β-carotene
	= 24 µg of other dietary provitamin A carotenoids (α-carotene or β-cryptoxanthin)

RAE=retinol activity equivalents

Recommended dietary allowance for vitamin A
The recommended dietary allowances (RDAs) for vitamin A are shown in Table 3, stratified according to age groups and sex.[18]

Table 3. Recommended dietary allowances (RDAs) for Vitamin A

Age	Male	Female	Pregnancy	Lactation
0–6 months*	400 µg RAE	400 µg RAE		
7–12 months*	500 µg RAE	500 µg RAE		
1–3 years	300 µg RAE	300 µg RAE		
4–8 years	400 µg RAE	400 µg RAE		
9–13 years	600 µg RAE	600 µg RAE		
14–18 years	900 µg RAE	700 µg RAE	750 µg RAE	1,200 µg RAE
19–50 years	900 µg RAE	700 µg RAE	770 µg RAE	1,300 µg RAE
51+ years	900 µg RAE	700 µg RAE		

*adequate Intake (AI), equivalent to the mean intake of vitamin A in healthy, breastfed infants.

Vitamin A as the treatment for COVID–19-related olfactory dysfunction
The dosage of the Carlson Vitamin A soft gels (7,500 µg RAE) is ~8 times the recommended dietary allowance (RDA) for VitA supplementation in an adult male.

Using the regimen of VitA treatment against measles infection, which consist of a total dose of 400,000 IU over two days [equivalent to 120,000 RAE (1 IU=0.3 µg RAE)], the daily supplementation of VitA 7,500 µg RAE can be given for 16 days before this therapeutic limit is exceeded.

This study proposed to give a 14-day course of daily oral VitA (7,500µg RAE) as the treatment for COVID–19-related OD, which equates to 105,000µg RAE (350,000 IU).

Contraindication(s) for vitamin A treatment
VitA treatment should not be used in pregnant women, especially during the first trimester, due to its teratogenic potential and increased risk of pregnancy complications such as spontaneous abortion and congenital foetal malformations.

Side-effect(s) of vitamin A treatment
Due to the naturally occurring nature of VitA in food substances (e.g., beef liver, sweet potato, spinach, etc.), a short-course treatment of VitA should be well tolerated.

Acute VitA toxicity has been reported previously, symptoms of hypervitaminosis A include nervous system disturbances, visual changes, and gastrointestinal discomforts.

On the other hand, chronic VitA over-supplementation is associated with osteoporosis and increased risk of hip fracture.

Treatment monitoring
Currently, serum monitoring of VitA level is not available in Queen Mary Hospital. However, serum bone markers such as calcium, phosphate, lactate dehydrogenase (LDH) and alkaline phosphatase (ALP) may be monitored periodically.

For female patients, prior to the initiation of vitamin A supplementation, the last menstrual period will be documented, and a urinary pregnancy test will be performed.

2.1.2.5 Resting-state functional magnetic resonance imaging (rs–fMRI) brain scans
OD is an important extra-pulmonary manifestation of COVID–19. Previous studies have illustrated the close relationship between olfactory, trigeminal, and gustatory functions in patients suffering from post-infectious anosmia.[19] However, the alterations of brain networks in COVID–19 patients are unknown. Therefore, we aim to study the networks related to olfactory, sensory and brain intrinsic activity, as these network regions may have decreased functional connectivity because of SARS-CoV-2 infection (Figure 1).

As network-based resting-state functional magnetic resonance imaging (rs-fMRI) of the brain is sensitive to functional cerebral changes, many recent studies have employed independent component analysis (ICA) and functional connectivity analyses to investigate the differences of anosmic patients before and after olfactory training.[8,20] Decreased connectivity was found in patients, and the connections of olfactory, somatosensory and integrative networks were modified after olfactory training.[20] Kollndorfer et al. also found the connectivity between piriform cortex and nonolfactory regions was reduced after olfactory training.[8] The neural reorganization processes of these three networks might be induced after smell training. The default mode (integrative) network (DMN) plays a major role in the studies of the brains in health and disease. Several studies found relationships of olfactory pathway and DMN.[21,22] Zhang and others found the intranasal insulin administration helped increase the connectivity between hippocampus and DMN regions in Type 2 diabetes patients.[23] Olfactory training might be capable of regaining the disturbed connections between olfactory network and DMN in COVID–19 patients.

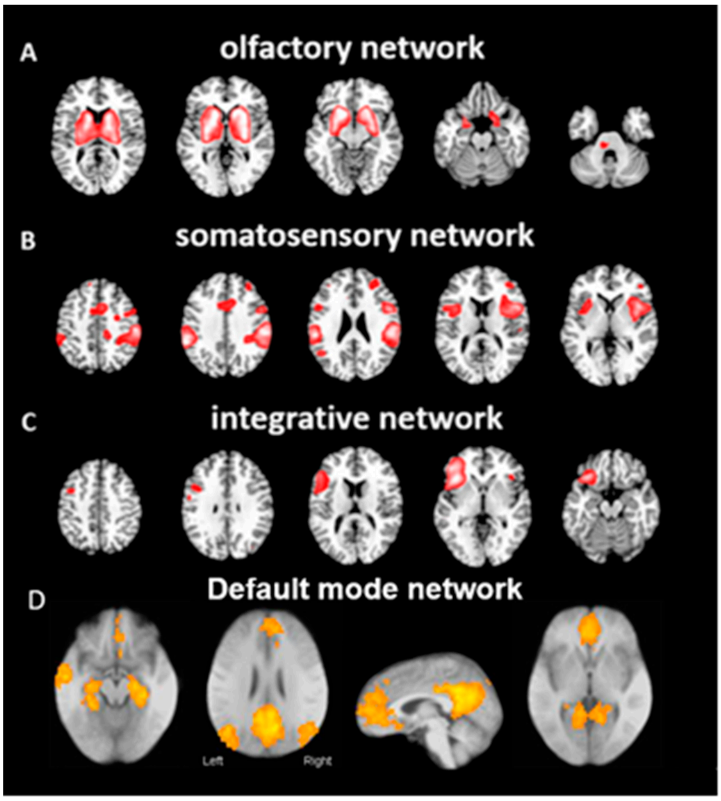

Figure 1. Anatomical images overlaid with (A) the olfactory network, (B) the somatosensory network, (C) the integrative network and (D) the default mode network. [Adapted with permission from Kollndorfer and others(2015a) and Ma et al. (2011)].

In addition, to investigate the neural underpinnings of the therapeutic and neurogenesis effect of oral VitA, the alterations between and within the olfactory, gustatory, somatosensory, and integrative networks will be assessed. We speculate that the connections between and within cerebral networks will be stronger in VitA plus OT group, when compared to OT alone.

3.1 Study hypothesis and objectives

3.1.1 Study hypothesis

A combination of oral vitamin A and intense aromatic chemosensory olfactory training (OT) by pulse aromatic stimulation will expedite the neurosensory recovery of olfaction in patients suffering from prolonged COVID–19-related OD.

3.1.2 Primary objectives

Objective differences in olfactory measurements between baseline and week 4 as documented by:

1. Butanol threshold test (BTT)
2. Smell identification test (SIT)

3.1.3 Secondary objectives

Documentation of objective modulations in the neuroradiological changes in the brain structures and cerebral network FC in the rs-fMRI brain scans between baseline, week 2, and week 4, as documented by:

1. Group independent component analyses and functional connectivity analyses of the olfactory, gustatory, somatosensory, and integrative networks before and after treatment in COVID–19 patients with OD
2. Group independent component analyses and functional connectivity analyses of the olfactory, gustatory, somatosensory, and integrative networks between combined VitA and OT versus OT alone in COVID–19 patients with OD
3. Group independent component analyses and functional connectivity analyses of the olfactory, gustatory, somatosensory, and integrative networks in the interventional versus control arms of COVID–19 patients with OD

3.2 Selection of study population

3.2.1 Inclusion criteria

1. Adult out-patient (≥18 years of age)
2. Previously diagnosed with coronavirus disease 2019 (COVID–19) by laboratory confirmation using reverse transcriptase polymerase chain reaction (RT-PCR) for the detection of severe acute respiratory virus coronavirus 2 (SARS-CoV-2)
3. Complaints of persistent olfactory disturbances, subjectively
4. Quantitatively documented to have olfactory dysfunction by
    a. Butanol threshold test (BTT)
    b. Smell identification test (SIT)
5. All subjects give written informed consent
6. Subjects must be available to complete the study and comply with study procedures.

3.2.2 Exclusion criteria

1. Inability to comprehend and to follow all required study procedures
2. Allergy or severe reactions to the study drug or smell training
3. Pregnant or breastfeeding women
4. Pre-existing factors which may account for persistent olfactory dysfunction besides COVID–19 (e.g., nasal polyps, obstructive lesions within the nasal cavity, severe anatomical malformations…)
5. Received an experimental agent (vaccine, drug, biologic, device, blood product, or medication) within 1 month prior to recruitment in this study or expect to receive an experimental agent during this study. Unwilling to refuse participation in another clinical study through the end of this study.
6. Have any condition that the investigator believes may interfere with successful completion of the study

3.3 Study design and organization

3.3.1 Overall study design

This is an open-labelled randomized-controlled trial (RCT) investigating the treatment safety and efficacy of oral vitamin A (VitA) in combination with olfactory training (OT) for patients suffering from prolonged COVID–19-related olfactory dysfunction (OD).

Prior to the initiation of treatment, all patients will receive subjective and objective olfactory assesessments. Comprehensive ear, nose, and throat (ENT) examination will be performed to rule out structural lesions which may account for persistent OD. All participants will receive resting-state functional magnetic resonance imaging (rs–fMRI) of the brain before treatment.

Patients with prolonged COVID–19-related OD will be assigned to the intervention (group A or B) or control (group C) arms:

Intervention arm

A. Short-course (14 days) daily oral VitA 25,000 international units (IU) in combination with OT three times per day for 4 weeks; or
B. OT three times per day for 4 weeks alone; or

Control arm

C. Clinical observation for 4 weeks

At the completion of the study, subjective and objective olfactory assesessments will be repeated to document clinical changes in olfaction. Follow-up rs-fMRI will be performed to document neuroradiological changes in the brain structures and cerebral network functional connectivies (FC).

3.2.2 Intervention and control

Intervention

1. Short-course (14 days) daily oral VitA 25,000 international units (IU) in combination with OT three times per day for 4 weeks; or
2. OT three times per day for 4 weeks alone; or

Control

3. Clinical observation for 4 weeks

3.3.3 Randomisation and blinding

This will be an open–label, randomised, controlled trial. Patients will be assigned to a serial number by the study-coordinator at PYNEH. Each serial number will be linked to a computer-generated randomisation list, which will assign treatment regimens by simple randomisation (1:1:0.5). The study medications will be dispensed by the hospital pharmacy. Participants and attending otolaryngologist will be aware of the group assignment. Neuroradiologists will be masked to the treatment allocations.

3.3.4 Withdrawal

Subjects may withdraw at any time without necessarily giving a reason and without prejudice. The investigator can also withdraw the subject for the following reasons:

1. Severe adverse events
2. Protocol violation
3. The attending physician considers that this is not in the best interest for the subjects to continue the study

3.4 Outcome measurements

3.4.1 Primary outcome measurements

Objective changes in olfactory measurements between baseline and week 4, as documented by:

1. Butanol threshold test (BTT)
2. Smell identification test (SIT)

3.4.2 Secondary outcome measurements

Documentation of objective modulations in the neuroradiological changes in the brain structures and cerebral network FC in the rs-fMRI brain scans between baseline, week 2, and week 4, as documented by:

1. Group independent component analyses and functional connectivity analyses of the olfactory, gustatory, somatosensory, and integrative networks before and after treatment in COVID–19 patients with OD

2. Group independent component analyses and functional connectivity analyses of the olfactory, gustatory, somatosensory, and integrative networks between combined VitA and OT versus OT alone in COVID–19 patients with OD
3. Group independent component analyses and functional connectivity analyses of the olfactory, gustatory, somatosensory, and integrative networks in the interventional versus control arms of COVID–19 patients with OD

4.1 Informed consent

The investigator or his/her representative will explain the nature of the study to the subjects and answer all questions by the subjects regarding this study. Prior to any study-related screening procedures being performed on the subject, the informed consent statement will be reviewed and signed and dated by the subjects and the person who administered the informed consent.

4.2 Confidentiality of data

4.2.1 Confidentiality of study data

The aims and content of this study, and the results thereof are confidential and are not to be transmitted to any third party in any form or fashion. All persons involved in the study are bound by this confidentiality clause.

4.2.2 Confidentiality of subject data

Permission for direct access to a subject's data will be sought in writing by the investigator and from the subject as part of the informed consent procedure. This gives permission to examine, analyse, verify, and reproduce any records and reports that are important to the evaluation of the study. Any party (e.g., domestic and foreign regulatory authorities, monitors and auditors) with direct access must take all reasonable precautions within the constraints of the applicable regulatory requirements to maintain the confidentiality of the subjects' identities and the Hospital Authority's proprietary information. It is the monitor's responsibility to verify that each subject has consented, in writing, to direct access.

4.2.3 Archive of data

The investigator must retain all study documentation pertaining to the conduct of the study at the study site for a period of at least 5 years.

4.3 Ethical and administrative endorsement

4.3.1 Administrative and ethical approval

The study will be approved by the Institutional Review Board (IRB) of the University of Hong Kong and Hospital Authority.

4.3.2 Study team

Principal investigators: design of the study, co-ordination, and assessment of subjects for study enrollment, report, and documentation of adverse events of study articles, data collection, analysis and writing up. Co-investigators: co-ordination and assessment of subjects for study enrollment, report, and documentation of adverse events of study articles, data collection and analysis.

5. References

1. Chan JF, Yuan S, Kok KH, et al. A familial cluster of pneumonia associated with the 2019 novel coronavirus indicating person-to-person transmission: a study of a family cluster. *Lancet* **2020**; 395:514-23.
2. World Health Organization. Coronavirus disease 2019 (COVID-19) Weekly Operational Update on COVID-19 9 September 2020. https://www.who.int/docs/default-source/coronaviruse/weekly-updates/wou-9-september-2020-cleared.pdf?sfvrsn=d39784f7_2 (accessed 14/09/2020).
3. Guan WJ, Ni ZY, Hu Y, et al. Clinical Characteristics of Coronavirus Disease 2019 in China. *N Engl J Med* **2020**; 382:1708-20.
4. Chung TW, Sridhar S, Zhang AJ, et al. Olfactory Dysfunction in Coronavirus Disease 2019 Patients: Observational Cohort Study and Systematic Review. *Open Forum Infect Dis*. **2020**;7:ofaa199.
5. Brann DH, Tsukahara T, Weinreb C, et al. Non-neuronal expression of SARS-CoV- 2 entry genes in the olfactory system suggests mechanisms underlying COVID-19-associated anosmia. *bioRxiv* **2020**. doi:10.1101/2020.03.25.009084.
6. Damm M, Pikart LK, Reimann H, et al. Olfactory training is helpful in postinfectious olfactory loss: a randomized, controlled, multicenter study. *Laryngoscope*. **2014**;124:826-831.

7. Langdon C, Lehrer E, Berenguer J, et al. Olfactory Training in Post-Traumatic Smell Impairment: Mild Improvement in Threshold Performances: Results from a Randomized Controlled Trial. *J Neurotrauma*. **2018**;35:2641-2652.
8. Kollndorfer K, Fischmeister FP, Kowalczyk K, et al. Olfactory training induces changes in regional functional connectivity in patients with long-term smell loss. *Neuroimage Clin*. **2015**;9:401-410.
9. Al Aïn S, Poupon D, Hétu S, Mercier N, Steffener J, Frasnelli J. Smell training improves olfactory function and alters brain structure. *Neuroimage*. **2019**;189:45-54.
10. Shearer KD, Stoney PN, Morgan PJ, McCaffery PJ. A vitamin for the brain. *Trends Neurosci*. **2012**;35:733-741.
11. Hummel T, Whitcroft KL, Rueter G, Haehner A. Intranasal vitamin A is beneficial in post-infectious olfactory loss. *Eur Arch Otorhinolaryngol*. **2017**;274:2819-2825.
12. World Health Organization. Measles. https://www.who.int/news-room/fact-sheets/detail/measles (accessed 22/06/2020).
13. Huiming Y, Chaomin W, Meng M. Vitamin A for treating measles in children. *Cochrane Database Syst Rev*. **2005**;2005:CD001479
14. Hussey GD, Klein M. A randomized, controlled trial of vitamin A in children with severe measles. *N Engl J Med*. **1990**;323:160-164.
15. Psaltis AJ, Li G, Vaezeafshar R, Cho KS, Hwang PH. Modification of the Lund-Kennedy endoscopic scoring system improves its reliability and correlation with patient-reported outcome measures. *Laryngoscope*. **2014**;124(10):2216-2223.
16. Paschaki M, Cammas L, Muta Y, et al. Retinoic acid regulates olfactory progenitor cell fate and differentiation. *Neural Dev*. **2013**;8:13.
17. Guidance for Industry: Converting Units of Measure for Folate, Niacin, and Vitamins A, D, and E on the Nutrition and Supplement Facts Labels. Docket Number: FDA-2016-D-4484. Center for Food Safety and Applied Nutrition.
18. Vitamin A Fact Sheet for Health Professionals. https://ods.od.nih.gov/factsheets/Vitamin%20A-HealthProfessional/#en5 Accessed on 15 June 2020.
19. Reichert JL, Schopf V. Olfactory Loss and Regain: Lessons for Neuroplasticity. Neuroscientist 2018;24:22-35.
20. Kollndorfer K, Kowalczyk K, Hoche E, et al. Recovery of olfactory function induces neuroplasticity effects in patients with smell loss. Neural Plast 2014;2014:140419.
21. Karunanayaka PR, Wilson DA, Tobia MJ, et al. Default mode network deactivation during odor-visual association. Hum Brain Mapp 2017;38:1125-39. 5.
22. Lu J, Yang QX, Zhang H, et al. Disruptions of the olfactory and default mode networks in Alzheimer's disease. Brain Behav 2019;9:e01296.
23. Zhang H, Hao Y, Manor B, et al. Intranasal insulin enhanced resting-state functional connectivity of hippocampal regions in type 2 diabetes. Diabetes 2015;64:1025-34.
